# Psychometric properties of Nursing Time Management Scale (NTMS), Arabic version

**DOI:** 10.1186/s12912-023-01316-7

**Published:** 2023-05-05

**Authors:** Raj’a Nayef Zyoud

**Affiliations:** Arab American University- Palestine, 13 Zababdeh, P.O Box 240, Jenin, Palestine

**Keywords:** Time management, Psychometrics, Nurses, Palestine, Factor analysis, Validity

## Abstract

Despite the presence of numerous generic time management instruments, relatively few research articles have assessed the validity and reliability of time management skills specific for the nursing profession. This study aimed at developing and validating a time management scale for nurses.

**Method** A self-administered questionnaire was administered to 715 nurses working in hospitals and clinics in the northern region of the West Bank, Palestine. The scale was examined through exploratory factor analysis, reliability measures, and correlations with other scales.

**Results** The scale revealed a 3-factor structure 1) organization of nursing work 2) planning and goal setting and 3) coordination of nursing work. The scale demonstrated excellent psychometric properties.

**Conclusions** The Nursing Time Management Scale (NTMS) is a valid and reliable measure that can be used in assessing time management skills of nurses and in evaluating interventions and training modules aiming at developing nurses’ time management skills.

**Supplementary Information:**

The online version contains supplementary material available at 10.1186/s12912-023-01316-7.

## Introduction

Time management is the process of organizing activities in chronological order with the aim of completing them in the most efficient way possible [1]. Efficiency means conducting the activities with the best quality and least costs. For people to efficiently manage their time, they should possess or develop certain skills such as planning, identifying priorities, becoming aware of the goals, objectives, creating an organized environment, and utilizing technologies that can automate or speed up activities.

Lacking time management skills creates problems in meeting deadlines, finishing tasks on time, and estimating the time needed to finish activities [2]. Consequently, interventions aiming at improving workers’ time management skills are crucial for improving services. To effectively implement time management interventions and evaluate their impacts, valid and reliable time management tools are needed.

Several time management instruments are available. The Assessment of Time Management Skills (ATMS) is a self-rated 30-item scale assessing organization, planning, and emotion regulation skills among people with executive functions deficiencies such as mental disorders. This scale was also found to be usable for the general population as well [1, 3]. The Time Organization and Participation Scale (TOPS) estimates time allocated to daily activities and time planning domains of time management [4]. The Zimbardo Time Perspective Inventory (ZTPI) assesses feelings related to different time perspectives [5]. The Time Management Behavior Scale (TMBS) comprises three subscales: (a) setting goals and priorities, (b) mechanics of time management, and (c) preference for organization among college students [6].

Despite the availability of time management instruments in the fields of education, psychology, and occupational therapy [2, 6, 7], few valid and reliable instruments are designed specifically for nurses. Nurses are the first-line responders to patients’ needs and their services are essential for patients’ healing and health. Improving nurses’ time management skills can have huge benefits for clinical work, efficiency, and organizational service quality. The aim of this study is to develop a questionnaire to measure time-management skills among nurses and to assess its psychometric properties.

A systemic review and meta-analysis of 13 articles concluded that a higher patient-to-nurse ratio, that is a nurse caring for more patients in a hospital is associated with an increase in needle stick injury, burnout, job dissatisfaction, and intent to leave [8]. Optimal nurse-to-patient ratios increase patient satisfaction and reduce nursing workload, burnout, fatigue, and decrease medication errors, pressure sores, and patient mortality [9]. It has been proposed that when a nurse cares for a lower number of patients there is an increase surveillance of patients’ symptoms, signs, and needs which alert hospital staff to potential problems upon which early actions can save lives [10]. The recommended nurse to patient ratio in a hospital varies by unit and shift from 1: 2 in intensive care and burn units, 1: 4 in intermediate care units, 1: 4 in morning shifts, 1: 5 in afternoon shifts, to 1: 8 in night shifts [11, 12]. Data on nurse-to-patient ratios are not available in Palestine but personal communications with nursing staff who work at local hospitals revealed that it is not uncommon for one nurse to care for at least 15 patients at a night shift.

A recently published article [13] reported lower nurse per 1000 people in Palestine compared to Israel and the Arab World and lower than the minimum WHO recommended 3 nurses per 1000 people [14], Table 1. This shortage of nurses highlights the importance of developing their time management skills to serve patients in the best way possible within the available resources.

**Table 1:** Number of hospitals, physicians and nurses /1000 people in Israel, Palestine, and the Arab World

	Israel	Palestine	Arab World
Hospital beds/1000 people	2.98	1.3	1.38
Physicians/1000 people	4.625	2.711	1.032
Nurses/1000 people	5.7	1.9	2.156

Source: [13]

### Questionnaire development

The questionnaire was developed based on an extensive review of the literature and previous time management questionnaires [2, 6, 7]. A total of 30 questions were included in the initial scale to measure various aspects of time management including goal setting, planning, scheduling, and organizing activities. Response categories were on a 5-point scale: 1) *never* 2) *infrequently* 3) *Sometimes* 4) *frequently* 5) *always*. The questionnaire also included demographic variables such as age, sex, experience, and previous attending a time management course.

### Data collection

Data were collected from 715 nurses working in 13 hospitals in the North of the West Bank of Palestine and several primary health care clinics. Data were collected between March and August 2019. Approval to collect the data was obtained from the Palestinian Ministry of Health. The researcher contacted the nursing directors of each hospital and primary healthcare center and asked them about the number of nurses working in their organization. Then the researcher printed out questionnaires according to the number and personally met with each nursing director, delivered the questionnaires to them and explained the data collection process including the content, objectives, and obtaining consent. Then the researcher asked the nursing directors to distribute the questionnaires to all the nurses in their organization, and to return the completed questionnaires to the nursing director within a week. Then the researcher personally collected the completed questionnaires from the nursing directors. The whole process was completed within 6 months. Data entry to SPSS version 25, was conducted by the researcher with the assistance of 2 experienced data entry personnel.

### Ethics approval and consent to participate

Nurses were told that completing the questionnaire is voluntary and no harms will result from their non-participation. They were also told that names will not be collected, and that data confidentiality will be ensured by keeping the questionnaires in a secure place used only for research purposes.

### Data analysis

Data analysis was conducted using SPSS version 25. The construct validity of the questionnaire and the underlying factors were explored through exploratory factor analysis. The internal consistencies of entire scale and the subdomains were measured through Cronbach’s Alpha in which a value between 0.70 and 0.95 is considered a good indicator of inter relatedness among the items [15]. Item-to-scale coefficients greater than 0.3 were considered appropriate [16, 17]. Items that did not meet these criteria were deleted from the final instrument.

Face and content validity were assessed through consulting with 10 experts in the fields of nursing and time management. The experts were informed about the aim of the study and the construct the scale is intended to measure. The experts were then asked to provide feedback on whether the items were clear and appear to be measuring what they are supposed to measure (face validity). The experts were also asked to provide feedback on whether the items were relevant to and represented the content domains of the intended construct. The author analyzed the feedback and revised the wording and number of items accordingly. The resulting questionnaire was tested on a pilot group of 5 staff nurses.

Concurrent and discriminant validity were assessed by correlating the NTMS with other scales that should be theoretically positively correlated (Brans habits), negatively correlated (Time Wasting Scale), or not correlated (Use of Technology Scale). The time wasting scale contained items like: I spend more time grooming than doing nursing work, I do not understand planning, I underestimate time needed to complete tasks, I cannot distinguish what is important from what is not. The use of technology scale contained items such as: I have internet access all day, I respond to different communication mediums (email, voice mail, others), and I have protected time to respond to sensitive phone- calls or emails. The Brans 12 virtues include: being honest with oneself, building trusting relationships, caring for one’s physical and mental health, and knowing how to schedule, plan, and prioritize activities [18].

## Results

Background characteristics of the participants are shown in Table 2. About 65.2% of the nurses were females and 34.8 males. The majority were above 35 years of age (43.9%) and held a bachelor degree (57.3%). Almost 72.6% worked in hospitals and 27.4% in outpatient clinics. Most worked in government institutions (75.1%) while 24.9% worked in private institutions. The majority of nurses (45.3%) had more than 10 years of job experience but most of them 42.5% had less than 5 years of management experience. Finally, a large percent of the participants (58.0%) attended a time management course.

**Table 2:** Background characteristics of the participants (*N* = 715)

Variable	Frequency	Percent
**Sex**		
Male	249	34.8
Female	466	65.2
**Age**		
Less than 25	201	28.1
Between 25–35	200	28.0
Above 35	314	43.9
**Education**		
Diploma	271	37.9
Bachelor	410	57.3
Master degree	34	4.8
**Organization**		
Hospital	519	72.6
Out- Patient Clinic	196	27.4
**Organization Type**		
Government	537	75.1
Private	178	24.9
**Job experience**		
Less than 5 years	225	31.5
Between 5–10 years	165	23.1
More than 10 years	324	45.3
**Management experience**		
Less than 5 years	304	42.5
Between 5–10 years	157	22.0
More than 10 years	210	29.4
**Attended a time management course**		
Yes	415	58.0
No	300	42.0
Total	715	100.0

From an original pool of 32 items, 3 were deleted after consulting with experts on face and content validity resulting in 29 items that were tested with factor analysis as described below.

Exploratory factor analysis was conducted on 30 time management items on the entire sample of 715 participants. This yielded 23.83 participants per item which is above the recommended minimum of 5 participants per item [19]. Factors were extracted using the principal components method based on Eigenvalues above one with Varimax rotation, mean replacement of missing values, and coefficients suppressed below 0.7. No item had missing values more than 5%.

**Fig. 1:**
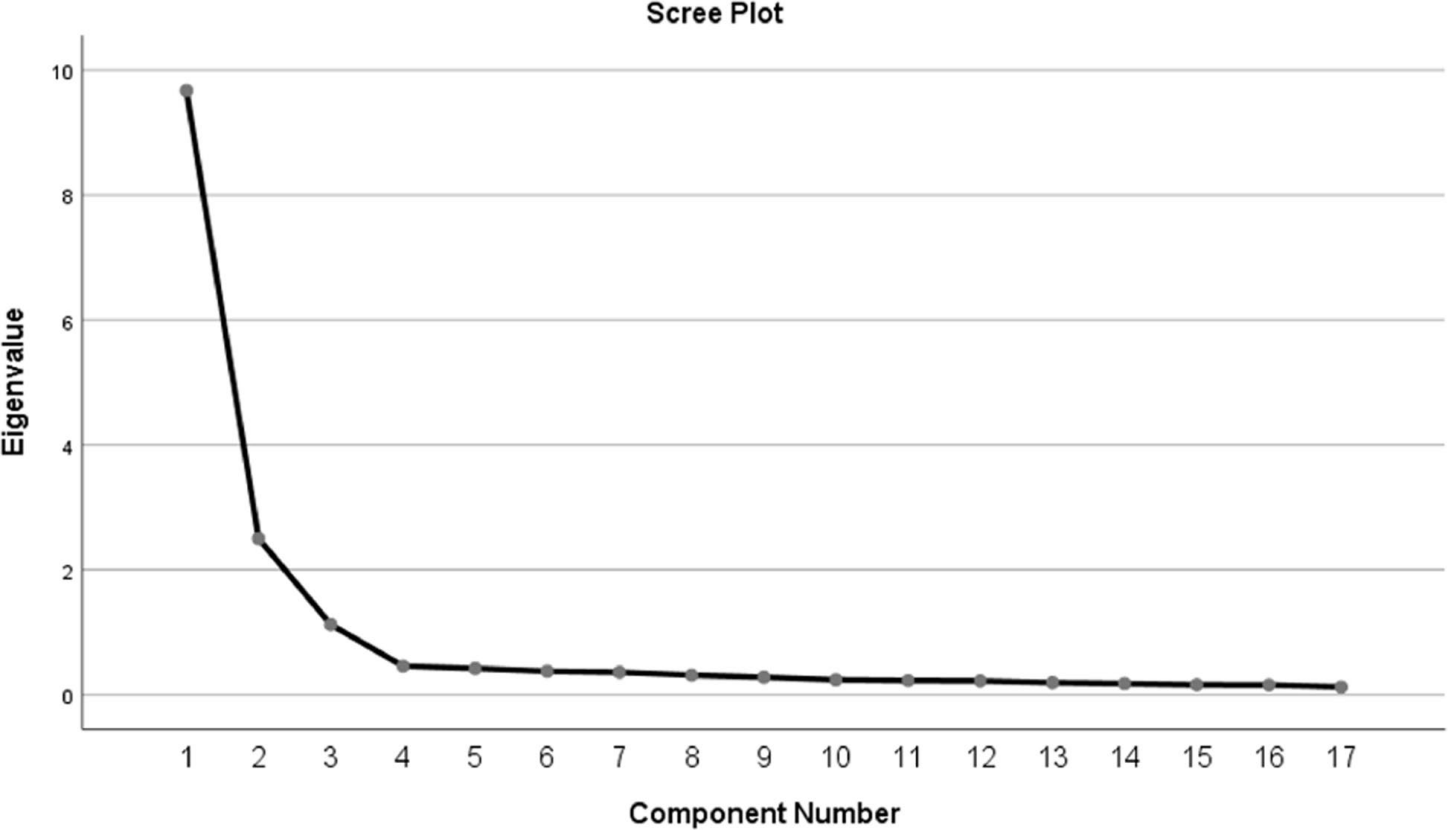
Scree plot showing the three-factor solution for the NTMS

The Kaiser–Meyer–Olkin (KMO) Measure of Sampling Adequacy was 0.950. This indicates strong partial correlations among the variables justifying the use of factor analysis. The Bartlett’s test of sphericity was < 0.001 rejecting the null hypothesis that the variables are not related i.e. the correlation matrix is not an identity matrix and justifying the use of factor analysis [20]. No item had a commonality value above 0.4 indicating the suitability of factor analysis for exploring the data. The scree plot in Fig. 1 shows the three extracted factors.

The factor loadings of each item are shown in Table 3. Twelve items were removed from a pool of 29 items due to weak or moderate loading (below 0.7) or cross loading on more than one factor. The number of items in the final scale was 17. The three factor structure accounted for 78.17% of the variance in the nursing time management scale. The first factor contained seven items on *organizing the workplace for nursing interventions* and accounted for 56.79% of the variation in the scale (Eigen value = 9.65). The second factor contained six items related to *setting goals and planning for activities.* It accounted for 14.73% of the variance (Eigen value = 2.50). The third factor contained four questions related to *coordination of activities* such as administering medications, treatments, procedures, and reports. The third factor accounted for 6.64% of the variance (Eigen value = 1.129).

**Table 3:** Factor loadings for each item from exploratory factor analysis (*N* = 715)

	Component		
	1 Organization	2 Planning	3 Coordination
I write a set of goals for myself for each day		0.820	
I have a set of goals for the entire week		0.855	
I force myself to make time for planning		0.781	
I spend enough time planning		0.879	
I have a time to think about how plans will be translated into action		0.819	
I have a clear idea of what I want to accomplish during day and make list of activities		0.751	
I Coordinate Medication Administration			0.817
I coordinate the administration of Treatments			0.861
I Coordinate the nursing Procedures			0.838
I Determine how reports will be given and received between shifts			0.754
I maintain a clean work area, and keep my desk organized	0.795		
I group activities that are in the same location	0.776		
I gather all equipment that will be needed before starting an activity	0.800		
I estimate the time needed to complete the task	0.809		
I document the nursing intervention as soon as possible after the activity is completed	0.814		
I handle paper work efficiently	0.789		
I utilize appropriate technology to facilitate communication and coordination	0.708		

Extraction Method: Principal Component Analysis

Rotation Method: Varimax with Kaiser Normalization

^a^Rotation converged in 6 iterations

### Discriminant and concurrent validity

According to Gefen and Straub (2005) [21], “discriminant validity is shown when the scale correlates weakly with other constructs that the scale is not theoretically related concurrent validity is demonstrated when two instruments measuring similar constructs are highly and significantly correlated.

Table 4 shows correlations between NTMS and other constructs. The NTMS correlated with other constructs in a theoretically expected manner. The Brans 12 habits are personal virtues related but not similar to time management skills [18]. The Brans 12 virtues scale was significantly positively correlated with the NTMS scale, correlation coefficient = 0.797, p < 0.001. On the other hand, the NTMS was negatively correlated with the time wasting scale correlation coefficient = -0.162, *p* < 0.001. Similarly, as expected, NTMS was not related to technology use scale. The scale also discriminated between those who attended a time management and those who did not.

**Table 4:** Correlations between the NTMS and other scales and variables (*N* = 715)

		**NTMS**	Brans	Time wasting scale	Technology use scale
**NTMS**	Pearson Correlation	**1**			
	Sig. (2-tailed)				
Brans	Pearson Correlation	**.797**^**b**^	1		
	Sig. (2-tailed)	**.000**			
Time wasting scale	Pearson Correlation	**-.162**^**b**^	-.136-^b^	1	
	Sig. (2-tailed)	**.000**	.000		
Technology use scale	Pearson Correlation	**.047**	-.007	.526^**^	1
	Sig. (2-tailed)	**.209**	.849	.000	
Attending Time Management Courses	Pearson Correlation	**-.140-**^**b**^	-.208-^b^	.048	.107^b^
	Sig. (2-tailed)	**.000**	.000	.203	.004

^b^Correlation is significant at the 0.01 level (2-tailed)

Table 5 shows that the NTMS mean for those who have attended a time management course (3.838) is higher than for those who have not attended such a course (3.586), F = 14.170, *p* < 0.001.

**Table 5:** Means of NTMS by having attended a time management course

	Mean	N	SD
yes	3.838	415	.91135
no	3.586	300	.83778
Total	3.732	715	.88934

## Reliability

The Cronbach’s Alpha for the 18-item scale was 0.953. For the 4-item coordination subscale, the Cronbach’s Alpha was 0.903. The Cronbach’s Alpha for the 7–item organization subscale was 0.950 and for the 6-item goals and planning subscale was 0.930. These results indicate excellent internal consistency for the whole and subscales.

### Instrument scoring

Responses to each item in the scale range from 1 indicating never practicing the time management skill to 5 indicating always practicing the activity. The best overall assessment of time management skills will be indicated with a summative score of all the 17 items. A minimum score of 17 will indicate the lowest time management skills. A score of 85 will be the highest score possible indicating advanced time management skills.

## Discussion

This study aimed at developing and evaluating a time management scale for Arabic speaking nurses. The results showed that NTMS is a valid and reliable scale that can be used to assess nurses’ time management skills. The three subscales identified with factor analysis were found to have excellent measurement properties. Those subscales were 1) organization of nursing activities and tasks (7 items) 2) planning activities and setting goals (6 items) 3) coordination of activities and procedures (4 items).

Maintaining a clean and organized space and instruments is critical for starting the planning, scheduling, and goal setting process. After the planning process is complete, the implementation phase commences which requires coordination of nursing activities with other team members. Administering medications, treatments, and procedures need to be well documented and shared with the other health team members to avoid medical errors. This scale assesses this important aspect of time management of coordinating for and organizing for nursing interventions.

The domains of this scale are similar to domains included in other time management scales but are more specific for the nursing profession (3, 4, 6). Therefore, this scale is more appropriate for research, projects, or programs aiming at assessing nurses’ time management skills. The scale can also be used in evaluating the effectiveness of interventions and training programs aiming at improving nurses’ time management skills. The scale can also be useful to raise awareness among nurses about their own time management skills and to identify weak areas that need improvement.

Currently there is growing evidence that time management interventions are effective in improving nurses’ organizational skills, decreasing work stress levels, and improving quality of work [22–25]. However, the scales used in these studies are either generic, author developed, and have not been extensively examined. The NTMS is a validated and reliable instrument designed specifically to measure time management skills in the nursing profession.

## Implications

This validated scale can be used by healthcare organizations to assess the time management skills of their nursing staff and take actions based on the results to improve the quality of their nursing care. This tool can also be used in assessing the effectiveness of interventions aiming at improving time management capacities of nurses. The scale can also be used for research purposes to examine strategies and factors influencing time management in the nursing profession. It is recommended that this scale be translated to other languages and validated in other contexts and settings.

## Limitations

Although this research was conducted on a representative sample in the northern areas of Palestine, generalizability to other contexts cannot be conclusively established. Further research in other contexts is needed to firmly establish the generalizability of the findings. The large sample size of this study, however, addresses major limitations of small sample sizes in previous studies. In addition, this research succeeded in reducing the number of items to 17 while keeping high measurement standards. However, the large sample size of the study addresses major limitations of small sample sizes in previous studies.

## Conclusions

The NTMS is a valid, reliable, and useful measure to assess and monitor improvements in time management skills among nurses. More research however is needed to examine the psychometric properties of this scale in other languages and contexts.

## Supplementary Information

**Additional file 1.**

## Data Availability

The datasets used and analysed during the current study available from the corresponding author on reasonable request.
