# Supplementary material for: Psychometric properties of Nursing Time Management Scale (NTMS), Arabic version
Source: BMC Nurs. 2023 May 5;22:153. doi: 10.1186/s12912-023-01316-7 (PMC10161497; doi:10.1186/s12912-023-01316-7)
Supplement: Supplementary file 1 — Additional file 1. [file 12912_2023_1316_MOESM1_ESM.docx]

| I write a set of goals for myself for each day |
| --- |
| I have a set of goals for the entire week |
| I force myself to make time for planning |
| I spend enough time planning |
| I have a time to think about how plans will be translated into action |
| I have a clear idea of what I want to accomplish during day and make list of activities |
| I coordinate the administration of Medication |
| I coordinate the administration of Treatments |
| I Coordinate the nursing Procedures |
| I Determine how reports will be given and received between shifts |
| I maintain a clean work area, and keep my desk organized |
| I group activities that are in the same location |
| I gather all equipment that will be needed before starting an activity. |
| I estimate the time needed to complete the task |
| I document the nursing intervention as soon as possible after the activity is completed |
| I handle paper work efficiently |
| I utilize appropriate technology to facilitate communication and documentation |
